# Kuntai Capsule plus Hormone Therapy vs. Hormone Therapy Alone in Patients with Premature Ovarian Failure: A Systematic Review and Meta-Analysis

**DOI:** 10.1155/2019/2085804

**Published:** 2019-06-26

**Authors:** Weiping Liu, Truong-Nam Nguyen, Thu-Van Tran Thi, Shaohu Zhou

**Affiliations:** ^1^The First Clinical College of Guangzhou University of Chinese Medicine, Guangzhou 510405, China; ^2^Vietnam University of Traditional Medicine, Hanoi 100000, Vietnam; ^3^The First Affiliated Hospital, Guangzhou University of Chinese Medicine, Guangzhou 510120, China

## Abstract

The aim of this study was to evaluate the efficacy and safety of Kuntai capsules (KTC) plus hormone therapy (HT) compared to HT alone for the treatment of premature ovarian failure (POF). Databases including PubMed, MEDLINE, Web of Science, China National Knowledge Infrastructure (CNKI), the Chinese BioMedical database (CBM), and the Wanfang database were searched up to October 2018 for randomized controlled trials (RCTs). After screening the studies, extracting the data, and assessing the study quality, Cochrane RevMan 5.3 software was used to conduct a meta-analysis. Twelve RCTs involving 1178 patients were included. Regarding the therapeutic effects, total effective treatment rate was higher for the KTC+HT groups compared to the HT-only groups. Furthermore, compared with HT, KTC+HR effectively altered endocrine indexes involving serum levels of luteinizing hormone (weighted mean difference [*WMD*]=-3.47, 95% CI [5.68, -1.26],* P*=0.002]), follicle-stimulating hormone [*WMD*=-8.15, 95% CI [-10.44, -5.86],* P*<0.00001], estrogen [*WMD*=17.21, 95% CI [10.16, 24.26],* P*<0.00001], and anti-Müllerian hormone [*WMD*=1.07, 95% CI [0.78, 1.36],* P*<0.00001]; blood lipid indexes involving serum levels of triglyceride (*WMD*=-0.55, 95% CI [-0.76, -0.43],* P*<0.00001), total cholesterol (*WMD*=-0.63, 95% CI [-0.74, -0.52],* P*<0.00001), and low-density lipoprotein cholesterol (*WMD*=-0.62, 95% CI [-0.75, -0.49],* P*<0.00001); and B-ultrasound results involving ovarian resistance index (*WMD*=-0.20, 95% CI [-0.35, -0.04],* P*=0.01), perfusion index (*WMD*=-0.41, 95% CI [-0.57, -0.24],* P*<0.00001), peak systolic velocity (*WMD*=2.43, 95% CI [1.52, 3.34],* P*<0.00001), antral follicle count (*WMD*=1.20, 95% CI [0.41, 2.00],* P*=0.003), and mean ovarian diameter in the plane containing the longest axis of the ovary (*WMD*=4.34, 95% CI [2.94, 5.74],* P*<0.00001). There were no serious adverse events in either group. There is evidence that KTC+HT is more effective and safer than HT alone for treating POF. However, the trials had low methodological quality and small samples, so further standardized research is required.

## 1. Introduction

Premature ovarian failure (POF), also known as premature ovarian insufficiency, refers to ovarian dysfunction in women aged <40 years. POF is included in the traditional Chinese medicine (TCM) categories [1] of infertility, late menstruation, and amenorrhea. It is characterized by anovulation and alterations of sex steroid hormone levels, including increased follicle-stimulating hormone (FSH) and luteinizing hormone (LH) levels and decreased estrogen (E2) levels. The main signs and symptoms of POF include tidal fever, night sweats, hot flushes with red facial skin, decreased sexual desire, vaginal dryness, atrophic genitalia, irregular or missed periods (amenorrhea), and difficulty conceiving or infertility. POF is a common cause of ovarian failure, and it occurs in 1% of women aged <40 and 0.1% of women aged <30 [2, 3]. POF is different from menopause, as intermittent and unpredictable ovarian function is found in approximately 50% of cases, and it can develop gradually over several years. Oocyte donation is currently the only way to help a patient with POF get pregnant [4]; however, this method can lead to tremendous emotional distress and financial burdens.

Currently, there are no specific treatments to cure POF. Symptomatic treatments, including HT, ovulation induction, immunosuppressive therapy, and oocyte donation, play key roles in the management of POF [5]. HT-based “add-back therapy” has been shown to improve the signs and symptoms of hypoestrogenism and prevent genital atrophy. However, long-term HT requires cautiously and continuously weighing up the benefits and risks for individual patients, and HT does not improve the follicular growth or ovulation rate and does not lead to recovery of the normal endocrine function of the ovaries [6–8]. Several studies have shown that long-term HT causes neurodegenerative diseases, hepatic damage, and vascular conditions such as venous thrombosis, and it increases the risk of endometrial, ovarian, and breast cancer [9–11]. Therefore, identifying effective and safe alternative medicines has become an urgent priority. Research has indicated that HT combined with traditional Chinese herbal preparations is more effective than HT alone, and interest in traditional Chinese herbal preparations, such as Kuntai capsules (KTC), is increasing [12].

TCM and other oriental medicine systems were established based on medical experiences over generations when Western medicine or modern medicine innovations were immature and underdeveloped. According to TCM theories, the pathogenesis underlying POF is caused by a deficiency of the kidney essence and the blood stored in the liver, making the ovaries unable to ensure conception, because of amenorrhea. Blood deficiency leads to Yin deficiency, which disturbs the heart (mind) due to hyperactivity of asthenic fire.

KTC is a form of TCM based on a modified Huanglian Ajiao decoction (which is a decoction containing* Rhizoma Coptidis* and* Donkey-hide Gelatin*). Huanglian Ajiao decoction is included in the publication* Shang Han Za Bing Lun *(*Treatise on Exogenous Febrile Diseases*), which was written by the eminent Chinese TCM physician, writer, and inventor Zhang Zhong Jing in the 3^rd^ century. KTC contains six components: Prepared Dihuang (*Radix Rehmanniae Praeparata*), Huanglian (*Rhizoma Coptidis*), Ajiao (*Donkey-hide Gelatin*), Baishao (*Radix Paeonia Alba*), Fuling (*Poria Cocos*), and Huangqin (*Radix Scutellaria Baicalensis*). It brings about the essential effects of clearing away the heat and tranquilizing the mind and is frequently prescribed with HR for POF patients because of its considerable therapeutic benefits.

However, the evidence regarding KTC remains controversial and there is currently no consensus on its benefit for the treatment of POF. Therefore, the aim of this systematic review and meta-analysis of clinical studies (which was conducted according to the criteria of the Cochrane Handbook [13]) was to determine the clinical efficacy and safety of the combination of KTC and HT compared to HT alone.

## 2. Materials and Methods

### 2.1. Search Strategy

An electronic search of PubMed, MEDLINE, Web of Science, China National Knowledge Infrastructure (CNKI), the Chinese BioMedical database (CBM), and the Wanfang database was conducted by two independent researchers. The search terms were as follows: “Kuntai capsule” OR “hormone therapy” AND “premature ovarian failure” OR “decreased ovarian reserve function” OR “POF” AND “efficacy” OR “effect”. The search was conducted up to October 2018.

### 2.2. Selection Criteria

#### 2.2.1. Inclusion Criteria

Studies were included if (1) study design was an RCT (with no restrictions regarding language, blinding, or publication type); (2) POF was diagnosed according to Chinese and international standards; (3) patients treated with KTC combined with HT were defined as the trial group; patients treated with HT alone were defined as the control group; (4) information about the therapeutic effects of KTC combined with HT was included; and (5) data could be extracted for a meta-analysis.

#### 2.2.2. Exclusion Criteria

Studies were eliminated if (1) study design was a non-RCT (without randomization or a control group), review, animal study, case report, report of specialist experiences, etc.; (2) publication was a duplicate article; (3) data could not be extracted; (4) sample size <30 cases; and (5) treatment measurements did not meet the predetermined inclusion criteria.

### 2.3. Study Selection and Data Analysis

To verify that the study was eligible for inclusion according to the inclusion and exclusion criteria, the titles, abstracts, and full texts were independently assessed by two researchers. If there was a disagreement between the two researchers during cross-checking, it was discussed with or dealt with by a third-party researcher. Data were extracted and tabulated, including the first author, year of publication, between-group comparability of baseline characteristics, sample size, intervention, duration of treatment, outcome measures and results, and adverse reactions.

### 2.4. Quality Assessment

The methodological quality of the included studies was independently evaluated by two researchers with reference to the Cochrane Handbook [13]. The evaluation included assessments of selection bias (random sequence generation and allocation concealment), performance bias (blinding of participants and personnel), detection bias (blinding of outcome assessments), attrition bias (incomplete outcome data), reporting bias (selective reporting), and other bias (other sources of bias). For each type of bias, each study was classified as having “low risk of bias”, “high risk of bias”, or “unclear risk of bias” and, according to the modified Jadad scale, the studies were classified as low quality (scores of 1–3) or high quality (scores of 4–7). If there was a disagreement during cross-checking, it was further discussed with or dealt with by a third-party researcher.

### 2.5. Statistical Analysis

The meta-analysis was performed using the RevMan 5.3 statistical software provided by the Cochrane Collaboration. For testing the between-study heterogeneity in the results, the *χ*^2^ test was used (significance level:* P*<0.1). If the heterogeneity test demonstrated no heterogeneity (*P≥*0.1, *I*^2^ < 50%), a fixed-effect model was used. A random-effects model was used if there was heterogeneity (*P*<0.1, *I*^2^ ≥ 50%); evidence of heterogeneity necessitated further subgroup analysis to determine the possible factors underlying the heterogeneity. Regarding the study outcomes, for binary variables, odds ratio (OR) or relative risk (RR) with 95% confidence interval (CI) were used, while for continuous variables, weighted mean difference (WMD) and 95% CI were used.* P*<0.05 indicated statistical significance. In addition, we planned to construct a funnel plot (to detect any publication bias) if the number of included studies was ≥9.

## 3. Results

### 3.1. Search Results

**Figure 1:**
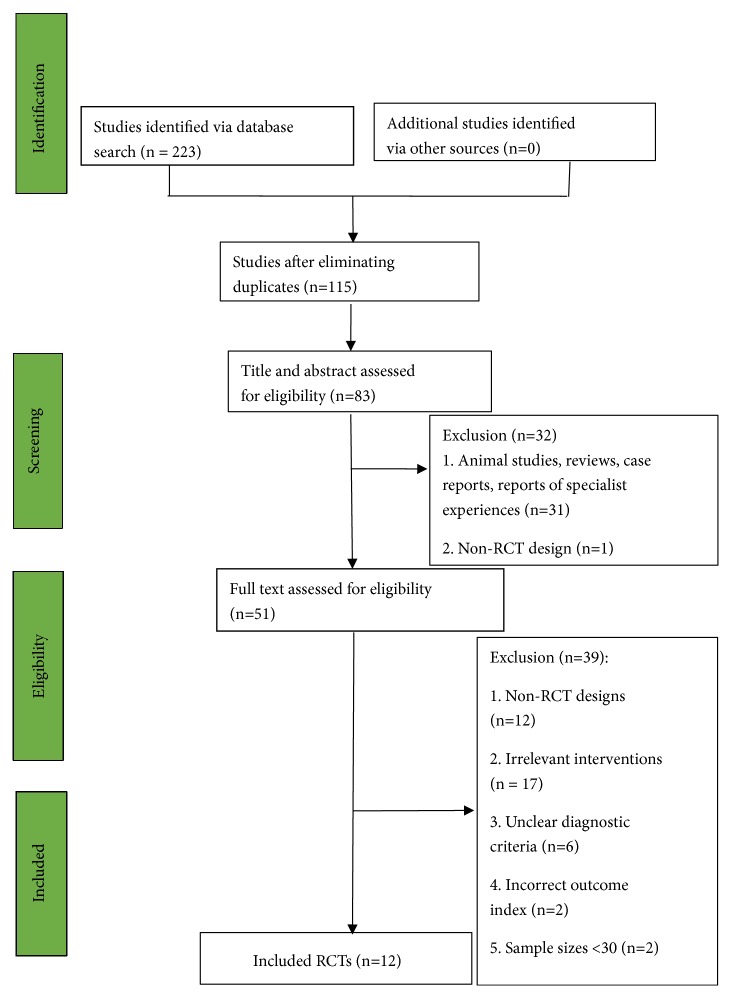
Flowchart of the search and selection process (n=223).

A flowchart of the search and selection process is shown in Figure 1. The systematic literature search retrieved 223 articles. After removal of duplicates, 115 articles remained. After a review of the titles and abstracts by two independent researchers, 32 studies were excluded (leaving 83) owing to irrelevance regarding the study topic. A further 32 studies were excluded due to being animal studies, reviews, case reports, reports of specialist experiences, and studies without randomization or a control group (non-RCT design), leaving 51 potentially eligible studies. After a review of the full texts, 39 studies were excluded due to non-RCT designs (without randomization or a control group), irrelevant interventions, unclear diagnostic criteria, incorrect outcome index, and sample sizes <30. This left 12 eligible RCTs [14–25] involving 1178 participants. All 12 were published in Chinese. The basic details of the studies are shown in Table 1.

### 3.2. Risk-of-Bias Assessment

**Figure 2:**
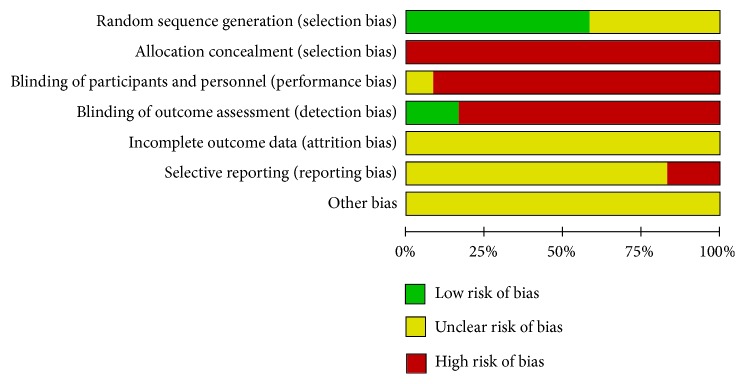
Risk of bias.

**Figure 3:**
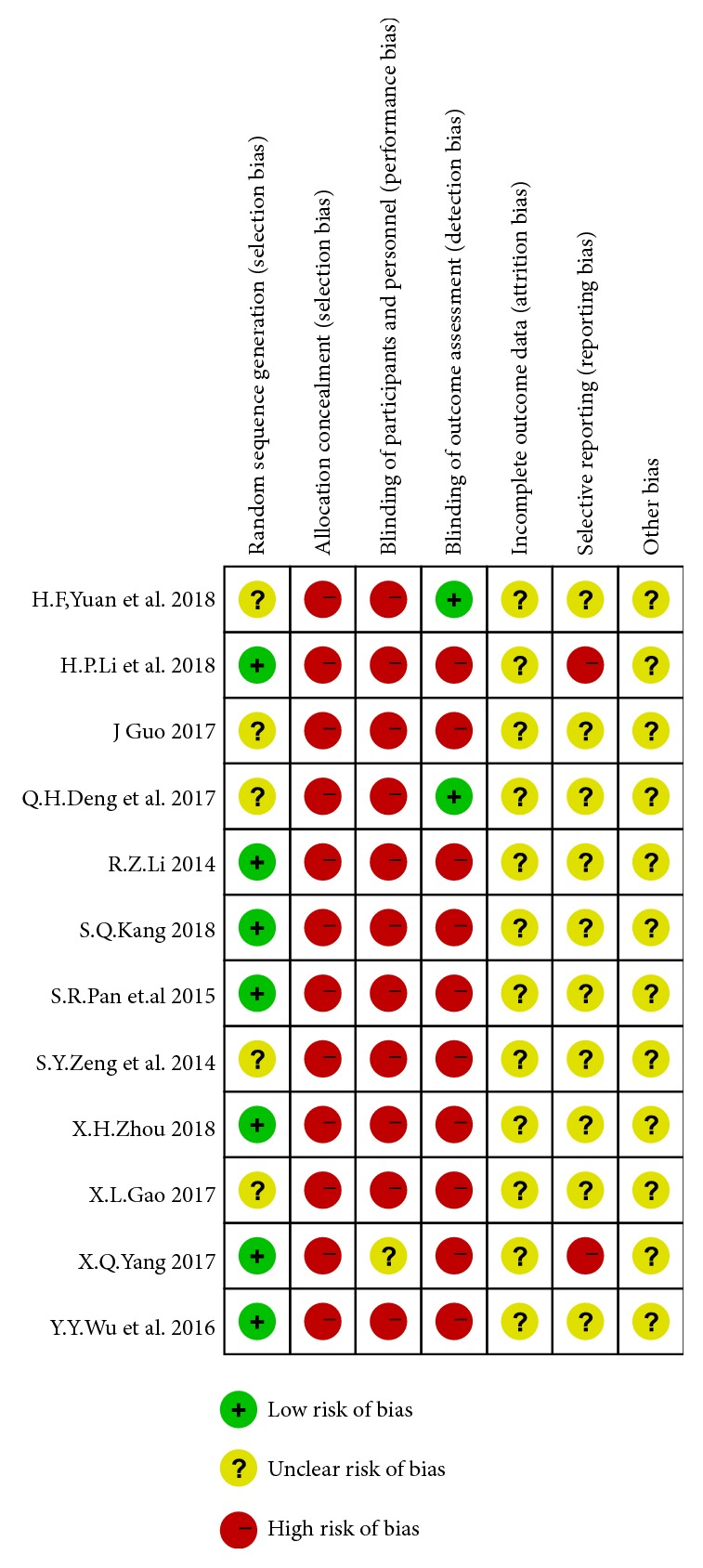
Risk-of-bias summary.

The risk-of-bias assessment of the included studies is shown in Figures 2 and 3.

### 3.3. Meta-Analysis Results

**Figure 4:**
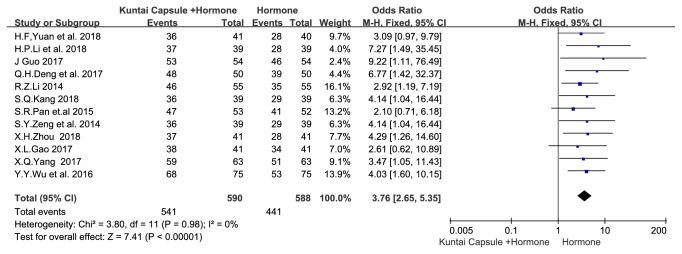
Meta-analysis results for the total effective treatment rate.

**Figure 5:**
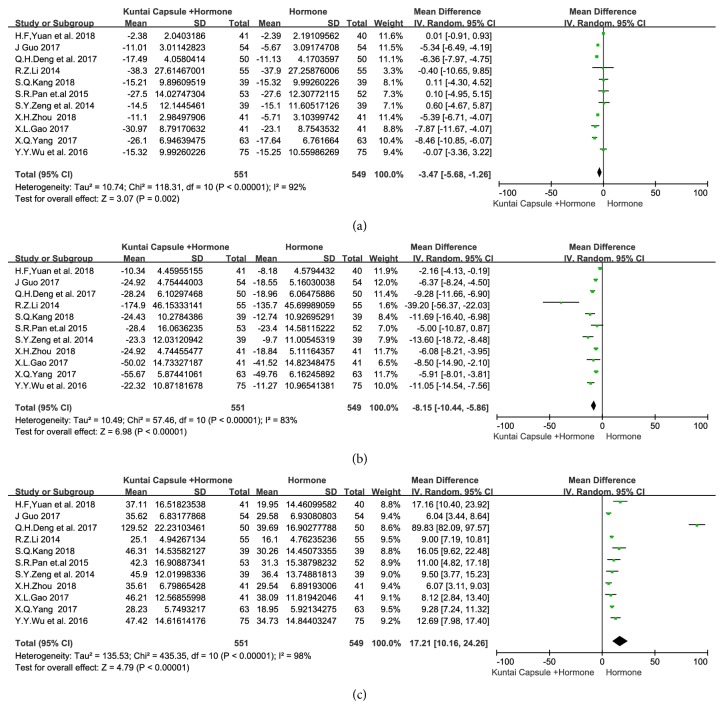
Meta-analysis results for the LH, FSH, and E2 levels. Note: (a) luteinizing hormone (LH); (b) follicle-stimulating hormone (FSH); and (c) estradiol (E2) serum levels.

**Figure 6:**
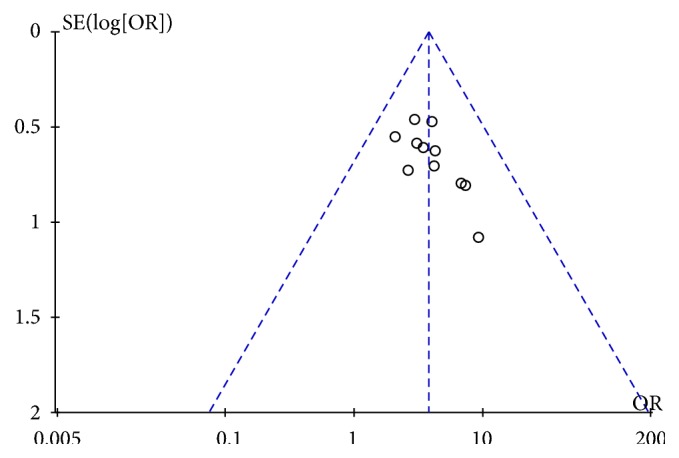
Funnel plot indicating a low risk of publication bias.

(3.3.1) A meta-analysis of the 12 articles showed that, for the total effective treatment rate, the homogeneity test results were* I*^*2*^=0% and P=0.98, indicating no significant heterogeneity. The fixed-effects meta-analysis demonstrated that the total effective treatment rate of KTC with HT was significantly better than that of HT alone (OR=3.76, 95% CI [2.65, 5.35],* P*<0.00001), as shown in Figure 4.

(3.3.2) Regarding the meta-analysis of the pre- and posttreatment serum levels of sex hormone comprising LH, FSH, and E2, 11 articles [14–17, 19–25] reported the pre- and posttreatment levels of LH, FSH, and E2. There was heterogeneity among the studies regarding the three outcomes (*I*^*2*^=92%,* P*<0.00001;* I*^*2*^=83%,* P*<0.00001;* I*^*2*^=98%,* P*<0.00001), so random-effects models were used. The meta-analysis showed that the serum levels of LH (weighted mean difference [*WMD*]=-3.47, 95% CI [-5.68, -1.26],* P*=0.002), FSH (*WMD*=-8.15, 95% CI [-10.44, -5.86],* P*<0.00001), and E2 (*WMD*=17.21, 95% CI [10.16, 24.26],* P*<0.00001) were significantly better in patients treated with KTC plus HT than in patients treated with HT alone, as shown in Figure 5.

(3.3.3) The meta-analysis results using (random-effects models) related to all other indexes are shown in Table 2. Compared to the control groups, the serum levels of anti-Müllerian hormone (AMH), triglyceride (TG), total cholesterol (TC), low-density lipoprotein cholesterol (LDL-C), ovarian resistance index (RI), perfusion index (PI), peak systolic velocity (PSV), antral follicle count (AFC), mean ovarian diameter (MOD) in the plane containing the longest axis of the ovary, and Kupperman score of the patients in the trial groups were significantly different (*P*<0.05). However, no significant difference in high-density lipoprotein cholesterol (HDL-C) was found between the trial and control groups.

(3.3.4) Publication Bias. As shown in Figure 6, the funnel plot of the included studies is roughly symmetrical, indicating a low risk of publication bias.

(3.3.5) Safety Evaluation. No severe adverse reactions were reported during the observation periods. As shown in Table 1, a safety evaluation was reported in five studies [14, 16, 19–21]. Three studies [16, 20, 21] reported that the difference in the incidence rate of adverse reactions between the two groups was not significant (*P*>0.05). The fourth study [14] reported that the incidence rate of adverse reactions in the control group was 21.95% (9/41) compared to 2.44% in the trial group (1/41), with a significant difference (*P*<0.05). The fifth study [19] reported that four patients in the control group had dizziness, which was relieved after HT withdrawal, while adverse reactions were not reported in the experimental group. The reported adverse reactions included nausea, vomiting, abdominal distension, other gastrointestinal symptoms, headache, dizziness, dysmenorrhea, breast tenderness, vaginal spotting, premenstrual syndrome, and abnormal hepatic function.

## 4. Discussion

POF is characterized by the absence of menarche or premature depletion of ovarian function among women aged <40 years. It is diagnosed based on sex hormone levels (including the serum levels of LH, FSH, E2, and AMH), B-ultrasound results (including RI, PI, PSV, AFC, and MOD) and Kupperman scores. These are all important criteria for evaluating ovarian reserve function and predicting ovarian function and pregnancy ability. It is noteworthy that KTC has been widely accepted in China for managing clinical and subclinical symptoms of ovarian failure [26, 27].

Among the ingredients of KTC,* Radix Rehmanniae Praeparata*, which is extracted by wine-steaming the roots of* Rehmanniae* (a plant belonging to the* Scrophulariaceae* family), is used as the monarch herb (i.e., the herb that plays the leading role in the treatment). It has been prescribed for several centuries as it can nourish the kidney-yin, improve the kidney essence, replenish the bone marrow, and tonify the blood (i.e., increase the available energy of the blood), based on the TCM theories mentioned in the publication* Bencao Tujing* (*Commentaries on the Illustrations*). This monarch herb is combined with minister herbs (i.e., herbs that reinforce the effect of the monarch herb and target auxiliary conditions or symptoms), including* Donkey-hide Gelatin*, which replenishes the vital essence and the blood, and* Radix Paeonia Alba*, which enriches the blood and calms the liver.* Rhizoma Coptidis* is especially effective at removing heat and dampness from the middle energizer while* Radix Scutellaria Baicalensis* mainly removes heat and dampness from the upper energizer. The compatibility of these two herbs means that, together, they act as adjuvant herbs, synergistically calming the mind and body. They are combined with* Poria cocos*, which invigorates the spleen and calms the mind. In combination, all of the components reinforce each other, which means that KTC supplements Yin and blood, clears away heat, nourishes the liver, tonifies the kidneys, soothes the spirit, moistens dryness, and regulates the benefits of Yin and Yang.

KTC has an estrogen-like effect and, combined with HT, it can improve ovarian function by regulating the hypothalamus-pituitary-ovary axis to promote follicular development and regulate the serum levels of sex hormones, thereby improving ovarian function [28]. More specifically, modern pharmacological research has shown that the effect of KTC on POF might be related to the estrogenic activity of* Radix Rehmanniae Praeparata* and* Radix Paeonia Alba *[29]. Research has indicated that KTC enhances the serum level of estrogen and leads to vaginal cell maturation index right-shifting [30]. Zhang et al. used menopausal animal models to show that KTC increases the ovarian volume, increases the uterine wet weight, recovers ovarian function, and tonifies the uterus [31]. Additionally, KTC can reduce the signs and symptoms of menopause in menopausal and postmenopausal patients, indicating that the effects of KTC might be related to the enhancement of ovarian function, and KTC does not have many adverse effects, despite having an estrogen-like effect [32]. Using real-time PCR and Western blot analysis, Cheng and Wang explored the molecular mechanisms of KTC and showed that it promotes the protein and peripheral serum mRNA levels of estrogen receptors *α* and *β* in perimenopausal patients [33]. Their research illustrated that KTC exerts its effects via multiple routes, not only by influencing the endocrine system. In summary, combined with HT, KTC can improve ovarian function by regulating the hypothalamus-pituitary-ovary axis to promote follicular development and regulate the serum levels of sex hormones, thereby improving ovarian function.

Based on the included studies (which all had Jadad scores of 1–2), the therapeutic benefits of KTC plus HT for the treatment of POF are significantly superior to the effects of HT alone. However, there is still a lack of high-quality international evidence and evidence from China. Meta-analysis is considered one of the most effective approaches to investigate the consistency of treatment effects across studies involving similar study populations. This study used meta-analysis to explore the therapeutic efficacy and safety of KTC plus HT for women with POF. The meta-analysis involved 12 RCTs with 1178 participants. It demonstrated that the total effective treatment rate of KTC plus HT was significantly higher than that of HT alone. Additionally, the endocrine indexes (including serum levels of LH, FSH, E2, and AMH), serum levels of lipids (including TG, TC, and LDL-C), B-ultrasound results (including RI, PI, PSV, AFC, and MOD), and Kupperman scores were clearly higher in the trial groups than the control groups. No serious adverse events were reported in the two groups.

There are several limitations in this study. First, the number of included studies and the total sample size are moderate; therefore, bias may have occurred due to inaccurate estimation based on the limited outcome data, which may have led to false positive results. Second, most of the included studies were not of excellent quality (all of the included studies had Jadad scores of 1–2), and they frequently lacked the data on randomization, blinding, and dropouts. Third, in the five safety evaluations, adverse reactions were observed during treatment and immediately after treatment, but there was a lack of long-term safety data. Therefore, more high-quality, large-sample, multicenter RCTs are needed to confirm the beneficial effects of KTC combined with conventional treatments on POF patients.

## Figures and Tables

**Table 1:** Basic details of included studies.

Included study	Sample size		Adverse reaction report	Interventions		Duration of treatment (month)	Outcome index	Jadad score
	Trial group	Control group		Trial group	Control group			
X.H. Zhou (2018)	41	41	Yes	Estrogen + Medroxyprogesterone + KTC	Estrogen + Medroxyprogesterone	3	A+B+C	2
Y.Y. Wu et al (2016)	75	75	NR	Climen (estradiolo valerato/cyproterone acetate) + KTC	Climen (estradiolo valerato/cyproterone acetate)	6	A+B	2
S.Q. Kang (2018)	39	39	Yes	Climen (estradiolo valerato/cyproterone acetate)/progesterone + KTC	Climen (estradiolo valerato/cyproterone acetate)/progesterone	6	A+B	2
S.Y. Zeng et al (2014)	39	39	NR	Progynova (estradiolo valerato)/progesterone + KTC	Progynova (estradiolo valerato)/progesterone	3	A+B	1
H.P. Li et al (2018)	39	39	NR	Progynova (estradiolo valerato)/progesterone + KTC	Progynova (estradiolo valerato)/progesterone	3 weeks	A+B+D	2
R.Z. Li (2014)	55	55	Yes	Progynova (estradiolo valerato) + KTC	Progynova (estradiolo valerato)	1	A+B	2
X.Q. Yang (2017)	63	63	Yes	Climen (estradiolo valerato/cyproterone acetate) + KTC	Climen (estradiolo valerato/cyproterone acetate)	6	A+B	2
S.R. Pan et al (2015)	53	52	Yes	Diethylstilbestrol /progesterone + KTC	Diethylstilbestrol /progesterone	6	A+B	2
H.F. Yuan et al (2018)	41	40	NR	Climen (estradiolo valerato/cyproterone acetate) + KTC	Climen (estradiolo valerato/cyproterone acetate)	3	A+B+E+F	1
Q.H. Deng e. al (2017)	50	50	NR	Yasmin (ethinylestradiol/drospirenone) + KTC	Yasmin (ethinylestradiol/drospirenone)	3	A+B+C +D+ E+G+H	1
J. Guo (2017)	54	54	NR	Progynova (estradiolo valerato) + KTC	Progynova (estradiolo valerato)	50 days	A+B+C	1
X.L. Gao (2017)	41	41	NR	Progynova (estradiolo valerato)/progesterone + KTC	Progynova (estradiolo valerato)/progesterone	3	A+B	1

Note: A: total effective treatment rate; B: luteinizing hormone (LH) + follicle-stimulating hormone (FSH) + estrogen (E2) levels; C: lipid index levels (triglyceride [TG], total cholesterol [TC], low-density lipoprotein-cholesterol [LDL-C], high-density lipoprotein-cholesterol [HDL-C]); D: ovarian resistance index (RI), perfusion index (PI), and peak systolic velocity (PSV); E: antral follicle count (AFC); F: anti-Müllerian hormone (AMH); G: Kupperman score; H: mean ovarian diameter (MOD) in the plane containing the longest axis of the ovary; NR: no report. In each study, all the baseline characteristics were equally distributed between the trial and control groups.

**Table 2:** Meta-analysis results for other indexes.

Index	Number of included studies	*I* ^*2 *^value (%)	*WMD*[95% CI]	*P* value
TG	3	0	−0.55[−0.67, −0.43]	<0.00001
TC	3	0	−0.63[−0.74, −0.52]	<0.00001
LDL-C	3	0	−0.62[−0.75, −0.49]	<0.00001
HDL-C	3	98	0.08[−0.47,0.63]	0.77
PI	2	0	−0.41[−0.57, −0.24]	<0.00001
RI	2	90	−0.20[−0.35, −0.04]	0.01
PSV	2	0	2.43[1.52,3.34]	<0.00001
AFC	2	90	1.20[0.41,2.00]	0.003
AMH	1	N/A	1.07[0.78,1.36]	<0.00001
Kupperman score	1	N/A	−5.99[−8.04, −3.94]	<0.00001
MOD	1	N/A	4.34[2.94,5.74]	<0.00001

Note: N/A: not available.
