# Corrigendum to “Kuntai Capsule plus Hormone Therapy vs. Hormone Therapy Alone in Patients with Premature Ovarian Failure: A Systematic Review and Meta-Analysis”

**DOI:** 10.1155/2021/5737914

**Published:** 2021-01-12

**Authors:** Weiping Liu, Truong-Nam Nguyen, Thu-Van Tran Thi, Shaohu Zhou

**Affiliations:** ^1^The First Clinical College of Guangzhou University of Chinese Medicine, Guangzhou 510405, China; ^2^Vietnam University of Traditional Medicine, Hanoi 100000, Vietnam; ^3^The First Affiliated Hospital, Guangzhou University of Chinese Medicine, Guangzhou 510120, China

In the article titled “Kuntai Capsule plus Hormone Therapy vs. Hormone Therapy Alone in Patients with Premature Ovarian Failure: A Systematic Review and Meta-Analysis” [1], errors have been identified in the forest plots (Figures 4 and 5).

The correct position of the *X*-axis labels, “Kuntai Capsule + Hormone” and “Hormone,” should be the opposite of their current position. The corrected Figures 4 and 5 are as follows.

## Figures and Tables

**Figure 1:**
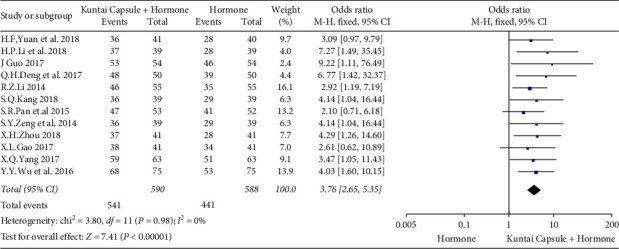
Meta-analysis results for the total effective treatment rate.

**Figure 2:**
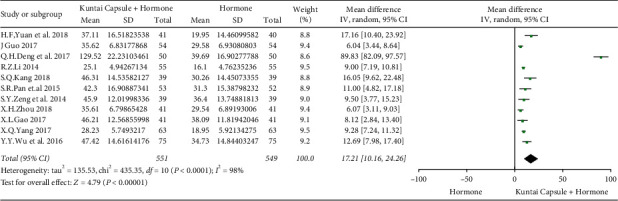
Meta-analysis results for the E2 levels.
